# Diminished Neutralization Capacity of SARS-CoV-2 Omicron BA.1 in Donor Plasma Collected from January to March 2021

**DOI:** 10.1128/spectrum.05256-22

**Published:** 2023-06-08

**Authors:** Yi-Chan J. Lin, David H. Evans, Ninette F. Robbins, Guillermo Orjuela, Kento T. Abe, Bhavisha Rathod, Karen Colwill, Anne-Claude Gingras, Ashleigh Tuite, Qi-Long Yi, Sheila F. O’Brien, Steven J. Drews

**Affiliations:** a Department of Medical Microbiology & Immunology, University of Alberta, Edmonton, Canada; b Scientific Affairs, Abbott Transfusion Medicine, Chicago, Illinois, USA; c Scientific Affairs, Abbott Transfusion Medicine, Bogotá, Colombia; d Lunenfeld-Tanenbaum Research Institute at Mt. Sinai Hospital, Sinai Health, Toronto, Ontario, Canada; e Department of Molecular Genetics, University of Toronto, Toronto, Ontario, Canada; f Dalla Lana School of Public Health, University of Toronto, Toronto, Ontario, Canada; g Epidemiology and Surveillance, Canadian Blood Services, Ottawa, Ontario, Canada; h School of Epidemiology and Public Health, University of Ottawa, Ottawa, Ontario, Canada; i Canadian Blood Services, Microbiology, Edmonton, Alberta, Canada; j Department of Laboratory Medicine and Pathology, University of Alberta, Edmonton, Alberta, Canada; Keck School of Medicine of the University of Southern California

**Keywords:** COVID-19 convalescent plasma, SARS-CoV-2 antibody, Omicron, neutralizing antibody, plaque reduction neutralization, method comparisons

## Abstract

The 50% plaque reduction neutralization assay (PRNT_50_) has been previously used to assess the neutralization capacity of donor plasma against wild-type and variant of concern (VOC) severe acute respiratory syndrome coronavirus 2 (SARS-CoV-2). Emerging data suggest that plasma with an anti-SARS-CoV-2 level of ≥2 × 10^4^ binding antibody units/mL (BAU/mL) protects against SARS-CoV-2 Omicron BA.1 infection. Specimens were collected using a cross-sectional random sampling approach. For PRNT_50_ studies, 63 previously analyzed specimens by PRNT_50_ versus SARS-CoV-2 wild-type, Alpha, Beta, Gamma, and Delta were analyzed by PRNT_50_ versus Omicron BA.1. The 63 specimens plus 4,390 specimens (randomly sampled regardless of serological evidence of infection) were also tested using the Abbott SARS-CoV-2 IgG II Quant assay (anti-spike [S]; Abbott, Chicago, IL, USA; Abbott Quant assay). In the vaccinated group, the percentages of specimens with any measurable PRNT_50_ versus wild-type or VOC were wild type (21/25 [84%]), Alpha (19/25 [76%]), Beta (18/25 [72%]), Gamma (13/25 [52%]), Delta (19/25 [76%]), and Omicron BA.1 (9/25 [36%]). In the unvaccinated group, the percentages of specimens with any measurable PRNT_50_ versus wild type or VOC were wild-type SARS-CoV-2 (16/39 [41%]), Alpha (16/39 [41%]), Beta (10/39 [26%]), Gamma (9/39 [23%]), Delta (16/39 [41%]), and Omicron BA.1 (0/39) (Fisher's exact tests, vaccinated versus unvaccinated for each variant, *P* < 0.05). None of the 4,453 specimens tested by the Abbott Quant assay had a binding capacity of ≥2 × 10^4^ BAU/mL. Vaccinated donors were more likely than unvaccinated donors to neutralize Omicron when assessed by a PRNT_50_ assay.

**IMPORTANCE** SARS-CoV-2 Omicron emergence occurred in Canada during the period from November 2021 to January 2022. This study assessed the ability of donor plasma collected earlier (January to March 2021) to generate any neutralizing capacity against Omicron BA.1 SARS-CoV-2. Vaccinated individuals, regardless of infection status, were more likely to neutralize Omicron BA.1 than unvaccinated individuals. This study then used a semiquantitative binding antibody assay to screen a larger number of specimens (4,453) for individual specimens that might have high-titer neutralizing capacity against Omicron BA.1. None of the 4,453 specimens tested by the semiquantitative SARS-CoV-2 assay had a binding capacity suggestive of a high-titer neutralizing capacity against Omicron BA.1. These data do not imply that Canadians lacked immunity to Omicron BA.1 during the study period. Immunity to SARS-CoV-2 is complex, and there is still no wide consensus on correlation of protection to SARS-CoV-2.

## INTRODUCTION

The use of convalescent plasma to treat patients infected with emerging respiratory viruses, including severe acute respiratory syndrome coronavirus 1 (SARS-CoV-1) and avian influenza, has been a topic of study for decades (1–3). Since the start of the SARS-CoV-2 pandemic, convalescent plasma was identified as a potential therapeutic candidate for clinical trials (4, 5). Those clinical trials identified mixed efficacy of convalescent plasma and the potential for early use of high-titer convalescent plasma in immunocompromised patients infected with SARS-CoV-2 (6–10). A revision of the U.S. Food and Drug Administration (FDA) emergency use authorization (EUA) for the use of COVID-19 convalescent plasma identified immunocompromised individuals as clinical trial candidates for high-titer COVID-19 convalescent plasma (11). Work on convalescent plasma has also led to further studies on protective immunity to SARS-CoV-2 (12–15) and has informed our incomplete understanding of the correlation of protection against SARS-CoV-2 (16, 17). Earlier convalescent plasma qualification approaches relied on low-throughput culture-based 50% plaque reduction neutralization (PRNT_50_) assays (18). Other, more rapid and easier-to-utilize approaches, including virus-like particle (VLP), competition assays, and enzyme-linked immunosorbent assays, were also used to identify high-titer plasma and study immune responses in individuals previously infected with SARS-CoV-2 (8, 12, 15, 19).

The Abbott SARS-CoV-2 IgG II Quant assay (Abbott anti-spike [S]; Abbott, Chicago, IL, USA; here referred to as the Abbott Quant assay) is a high-throughput assay that is simpler to operationalize than PRNT_50_. This assay generates semiquantitative results which can be converted into binding antibody units [BAU] per milliliter (20). A prior study noted that a cutoff of 7.1 × 10^3^ BAU/mL might be used to screen for neutralizing high-titer plasma against wild-type, Alpha, Beta, Gamma, and Delta SARS-CoV-2 (12). High-throughput semiquantitative technologies enable researchers to screen large numbers of plasma donations for unique specimens that might contain high-titer anti-SARS-CoV-2 neutralizing plasma against wild-type and variant of concern (VOC) SARS-CoV-2 (21).

SARS-CoV-2 VOC Omicron has shown an ability to partially evade both infection and vaccine-generated pre-Omicron neutralizing antibody capacity (22–25). In individuals with a prior BA.1 or BA.2 infection, there is also a marked decrease in neutralizing capacity against BA.2.12.1, BA.4, and BA.5 (26). Compared to BA.5, Omicron BQ.1.1 and XBB.1 subvariants were more likely to escape neutralizing antibodies after both monovalent and bivalent mRNA vaccine boosting (27). There is growing evidence that screening plasma using high-throughput immunosorbent assays at a threshold of ≥2 × 10^4^ BAU/mL may identify high-titer neutralizing plasma against Omicron BA.1 that could then be used in convalescent plasma clinical trials (28–30).

Assessments of neutralizing capacity of plasma or serum may be impacted by local and temporal factors. Prior to the emergence of Omicron, less than 10% of Canadians were estimated to have been naturally infected with SARS-CoV-2 (31, 32). Until January to March 2021, most infections in Canada were likely due to wild-type or Alpha SARS-CoV-2 (33). Vaccination campaigns were initiated in December 2020, with 96% of all Canadian blood donors showing evidence of measurable antibodies to anti-spike (S) by August 2021 (34). Canadian Blood Services was able to determine donor vaccination status most effectively for the time from January to March 2021 (12, 13).

This study used PRNT_50_ to determine the neutralizing capacity of vaccinated and unvaccinated donor plasma collected from January to March 2021against Omicron BA.1. This study also used the Abbott Quant assay to screen a larger number of donor plasma specimens collected from this time period for individual specimens potentially containing high-titer neutralizing capacity against Omicron BA.1.

## RESULTS

### All specimens were from donors with an anti-S or an anti-RBD serological signal.

Study specimens were subsamples of a larger repeated cross-sectional design with random cross-sectional sampling. Previously, 65 specimens were analyzed by PRNT_50_ (wild-type, Alpha, Beta, Gamma, and Delta SARS-CoV-2) (13) as well as the Abbott Quant assay (12). All specimens previously tested by PRNT_50_ had evidence of an anti-S or anti-receptor binding domain (RBD) signal (with or without anti-N) (12). Sixty-three specimens had sufficient sample volume to be tested by PRNT_50_ for Omicron SARS-CoV-2. For the 63 specimens, anti-N profiles, Abbott Quant assay results, donor vaccination histories, and PRNT_50_ (wild type, Alpha, Beta, Gamma, Delta, and Omicron SARS-CoV-2) are presented in Tables 1 to 4.

**TABLE 1:** Summary of Abbott Quant assay and PRNT_50_ results for vaccinated donors with anti-N signals (*n* = 5)^*a*^

Specimen no.	Vaccination history	PRNT_50_ against variant:						Abbott anti-S (BAU/mL)
		Wild type	Alpha	Beta	Gamma	Delta	Omicron	
CIHR013654	1 dose (≥14 days)	40	<20	<20	<20	<20	<20	2 × 10^2^
CIHR015946	Dose and timing NA	20	<20	<20	<20	<20	<20	7 × 10^1^
CIHR016894	Dose and timing NA	5,120	2,560	5,120	5,120	5,120	160	7 × 10^3^
CIHR017333	Dose and timing NA	1,280	640	160	160	160	40	3 × 10^3^
CIHR017730	Dose and timing NA	2,560	1,280	1,280	2,560	1,280	80	4 × 10^3^
Total donations with any neutralizing capacity (no. [%])		5 (100)	3 (60)	3 (60)	3 (60)	3 (60)	3 (60)	5 (100)

a Median Abbott Quant assay values for this group were 3 × 10^3^ BAU/mL (25th percentile to 75th percentile, 1 × 10^2^ to 6 × 10^3^ BAU/mL). NA, not available.

### Neutralization of wild-type and VOC SARS-CoV-2 in vaccinated versus unvaccinated donors.

Since different cell lines were used to understand the neutralizing capacity of donor plasma against Omicron SARS-CoV-2, median PRNT_50_ results were not compared directly. Instead, the numbers of specimens producing any neutralizing antibodies (e.g., ≥20) were compared within vaccinated and unvaccinated groups.

Small numbers of specimens for individuals with a vaccine history and an anti-N signal (possible evidence of a past SARS-CoV-2 infection) led to the combination of data from donors vaccinated with an anti-N signal (Table 1) and donors vaccinated without an anti-N signal (Table 2). Data from unvaccinated donors with an anti-N signal (Table 3) and unvaccinated donors without an anti-N signal (Table 4) were also combined.

**TABLE 2:** Summary of Abbott Quant assay and PRNT_50_ results for vaccinated donors without anti-N signals (*n* = 20)^*a*^

Specimen no.	Vaccination history	PRNT_50_ against variant:						Abbott anti-S (BAU/mL)
		Wild type	Alpha	Beta	Gamma	Delta	Omicron	
CIHR013818	Dose and timing NA	40	80	<20	<20	80	<20	2 × 10^2^
CIHR014329	Dose and timing NA	40	20	40	<20	40	<20	3 × 10^2^
CIHR015234	1 dose (≥14 days)	<20	<20	<20	<20	<20	<20	1 × 10^2^
CIHR015533	1 dose (≥14 days)	640	640	320	160	640	20	7 × 10^3^
CIHR015657	Dose and timing NA	40	20	20	<20	20	<20	8 × 10^2^
CIHR015884	Fully vaccinated	320	320	160	160	160	<20	2 × 10^3^
CIHR015958	Dose and timing NA	40	40	20	<20	20	<20	5 × 10^2^
CIHR016698	1 dose (≥14 days)	320	320	80	160	80	<20	2 × 10^3^
CIHR016904	Dose and timing NA	20	20	<20	<20	20	<20	2 × 10^1^
CIHR016905	Fully vaccinated	320	640	160	160	160	20	3 × 10^3^
CIHR016930	1 dose (≥14 days)	<20	<20	<20	<20	20	<20	0
CIHR017087	Dose and timing NA	80	80	80	20	80	<20	5 × 10^2^
CIHR017189	1 dose (≥14 days)	160	80	80	80	80	<20	1 × 10^3^
CIHR017229	Dose and timing NA	<20	<20	20	<20	<20	<20	1 × 10^1^
CIHR017534	Dose and timing NA	<20	<20	<20	<20	<20	<20	5 × 10^1^
CIHR017540	Dose and timing NA	80	40	20	<20	<20	20	4 × 10^2^
CIHR017728	Dose and timing NA	1,280	640	320	160	640	80	4 × 10^3^
CIHR017824	Dose and timing NA	160	160	160	80	80	<20	7 × 10^2^
CIHR017838	Dose and timing NA	640	640	640	320	640	40	3 × 10^3^
CIHR018126	Dose and timing NA	80	80	80	20	40	20	6 × 10^2^
Total donations with any neutralizing capacity (no. [%])		16 (80)	16 (80)	15 (75)	10 (50)	16 (80)	6 (30)	19 (95)

a Median Abbott Quant assay values for this group were 5 × 10^2^ BAU/mL (25th percentile to 75th percentile, 1 × 10^2^ to 2× 10^3^). NA, not available.

**TABLE 3:** Summary of Abbott Quant assay and PRNT_50_ results for nonvaccinated donors with anti-N signals (*n* = 19)^*a*^

Specimen no.	PRNT_50_ against variant:						Abbott anti-S (BAU/mL)
	Wild type	Alpha	Beta	Gamma	Delta	Omicron	
CIHR013757	<20	<20	<20	<20	<20	<20	2 × 10^0^
CIHR013936	80	40	40	40	40	<20	7 × 10^1^
CIHR014110	80	20	40	40	40	<20	2 × 10^2^
CIHR014113	80	20	20	40	40	<20	2 × 10^2^
CIHR014235	160	40	<20	<20	320	<20	3 × 10^2^
CIHR014309	80	40	20	<20	80	<20	3 × 10^2^
CIHR014840	40	<20	<20	<20	20	<20	1 × 10^2^
CIHR014884	160	160	<20	80	80	<20	2 × 10^2^
CIHR014993	<20	<20	<20	<20	<20	<20	1 × 10^1^
CIHR015094	320	320	640	80	640	<20	5 × 10^1^
CIHR015434	40	40	<20	<20	40	<20	6 × 10^1^
CIHR016024	20	20	<20	<20	<20	<20	7 × 10^1^
CIHR016624	20	20	40	20	20	<20	2 × 10^1^
CIHR016979	40	40	20	80	80	<20	1 × 10^2^
CIHR017127	<20	20	<20	<20	40	<20	2 × 10^1^
CIHR017305	80	40	20	<20	80	<20	1 × 10^2^
CIHR017724	160	80	80	80	160	<20	3 × 10^2^
CIHR017894	40	20	<20	<20	20	<20	5 × 10^1^
CIHR017990	80	40	20	20	40	<20	8 × 10^1^
Total donations with any neutralizing capacity (no. [%])	16 (84)	16 (84)	10 (53)	9 (47)	16 (84)	0	19 (100)

a Median Abbott Quant assay values for this group were 8 × 10^1^ BAU/mL (25th percentile to 75th percentile, 5 × 10^1^ to 2 × 10^2^).

**TABLE 4:** Summary of Abbott Quant assay and PRNT_50_ results for nonvaccinated donors without anti-N signals (*n* = 19)^*a*^

Specimen no.	PRNT_50_ against variant:						Abbott anti-S (BAU/mL)
	Wuhan	Alpha	Beta	Gamma	Delta	Omicron	
CIHR014238	<20	<20	<20	<20	<20	<20	0
CIHR014491	<20	<20	<20	<20	<20	<20	0
CIHR014632	<20	<20	<20	<20	<20	<20	1 × 10^1^
CIHR014664	<20	<20	<20	<20	<20	<20	0
CIHR014926	<20	<20	<20	<20	<20	<20	0
CIHR015079	<20	<20	<20	<20	<20	<20	0
CIHR015475	<20	<20	<20	<20	<20	<20	0
CIHR015843	<20	<20	<20	<20	<20	<20	0
CIHR015948	<20	<20	<20	<20	<20	<20	0
CIHR016403	<20	<20	<20	<20	<20	<20	0
CIHR016447	<20	<20	<20	<20	<20	<20	0
CIHR016548	<20	<20	<20	<20	<20	<20	2 × 10^1^
CIHR016557	<20	<20	<20	<20	<20	<20	0
CIHR016973	<20	<20	<20	<20	<20	<20	0
CIHR017530	<20	<20	<20	<20	<20	<20	0
CIHR017945	<20	<20	<20	<20	<20	<20	0
CIHR018000	<20	<20	<20	<20	<20	<20	1.4 × 10^1^
CIHR018166	<20	<20	<20	<20	<20	<20	1 × 10^0^
CIHR018178	<20	<20	<20	<20	<20	<20	0
Total no. of donations with any neutralizing capacity		0	0	0	0	0	4 (21)

a Median Abbott Quant assay values for this group were 0 BAU/mL (25th percentile to 75th percentile, 0 to 1 × 10^1^).

When neutralizing capacity was measured by PRNT_50_, plasma from vaccinated donors was more likely than plasma from unvaccinated donors to neutralize VOCs (including Omicron BA.1) and wild type. For wild-type neutralization, the proportions were vaccinated (21/25 [84%]) versus unvaccinated (16/39 [41%]) (*P* = 0.0008; odds ratio, 7.55; 95% confidence interval [CI], 2.31 to 22.67). For Alpha neutralization, the proportions were vaccinated (19/25 [76%]) versus unvaccinated (16/39 [41%]) (*P* = 0.01; odds ratio, 4.55; 95% CI, 1.42 to 14.80). For Beta neutralization, the proportions were vaccinated (18/25 [72%]) versus unvaccinated (10/39 [26%]) (*P* = 0.0003; odds ratio, 7.46; 95% CI, 2.25 to 23.02). For Gamma neutralization, the proportions were vaccinated (13/25 [52%]) versus unvaccinated (9/39 [23%]) (*P* = 0.03; odds ratio, 3.61; 95% CI, 1.29 to 10.03). For Delta neutralization, the proportions were vaccinated (19/25 [76%]) versus unvaccinated (16/39 [41%]) (*P* = 0.01; odds ratio, 4.55; 95% CI, 1.42 to 14.80). For Omicron (BA.1) neutralization, the proportions were vaccinated (9/25 [36%]) versus unvaccinated (0/39) (*P* ≤ 0.0001; odds ratio, ∞).

### Assessment of residual specimens from January, February, and March 2021 using the Abbott Quant assay.

**FIG 1:**
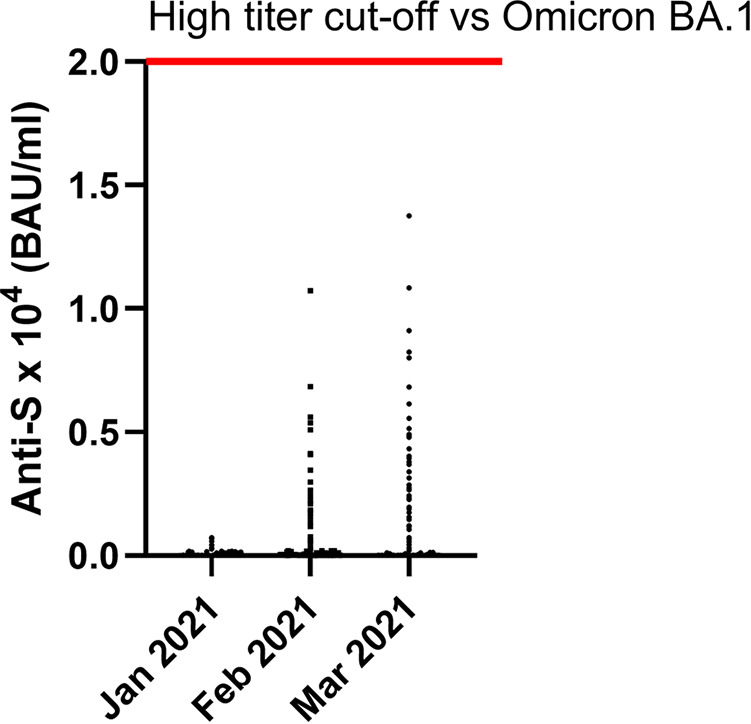
Anti-S BAU/mL levels for Canadian blood donors (April 2020 to March 2021). For this study, 4,453 retention specimens were available from January (*n* = 1,499), February (*n* = 1,465), and March (*n* = 1,489) for testing using the Abbott Quant assay. Anti-S BAU per milliliter values are on the *y* axis. Data are presented as scatterplots of BAU per milliliter values for each specimen monthly. The red line indicates a high-titer cutoff against SARS-CoV-2 Omicron BA.1 (≥2 × 10^4^ BAU/mL). None of the 4,453 plasma specimens contained an anti-S BAU/mL value of ≥2 × 10^4^ BAU/mL.

In addition to the 63 specimens tested by PRNT_50_ for Omicron and the Abbott Quant Assay, 4,390 randomly sampled specimens were tested by the Abbott Quant assay (*n* = 4,453). The monthly distribution of these 4,453 specimens collected in 2021 was 1,499 in January, 1,465 in February, and 1,489 in March. The BAU per milliliter values of these 4,453 specimens are presented in Fig. 1. None of the BAU per milliliter values reached a level of 2 × 10^4^ BAU/mL.

## DISCUSSION

For the period from January to March 2021, plasma collected from vaccinated Canadian blood donors was more likely to have measurable neutralizing antibodies (measured by PRNT_50_ against wild type, Alpha, Beta, Gamma, Delta, and Omicron BA.1) than plasma from unvaccinated blood donors. In the unvaccinated group, none of the plasma specimens had measurable PRNT_50_ titers versus Omicron BA.1. As previously noted, specimens were collected when seroprevalence to SARS-CoV-2 was <10% and when most Canadians with a history of SARS-CoV-2 infection would have been infected with wild-type or Alpha SARS-CoV-2 (32, 33). Only a minority (8%) of vaccinated donors in this study claimed to be fully vaccinated (12, 32), and only 2% of Canadians had received two doses of a SARS-CoV-2 vaccine (35). Wastewater studies and clinical specimens suggest that Omicron emergence occurred much later in Canada, during the period from November 2021 to January 2022 (36–40).

As previously described, Omicron BA.1 can escape neutralization from patients infected with non-Omicron strains. These trends are independent of specific geographic regions. In the United States, convalescent-phase serum collected from a small number of patients infected with Delta (*n* = 19) had lower levels of pseudovirus neutralization against BA.1 than convalescent-phase serum from BA.1-infected patients (*n* = 31) (41). In another U.S. study, postinfection serum panels (1 month postinfection [*n* = 64] and 6 months postinfection [*n* = 36]) collected prior to the emergence of BA.1 exhibited decreased neutralization against BA.1 than wild-type SARS-CoV-2 when measured with a 50% fluorescent focus reduction neutralization titer (FFRNT_50_) assay (42). Convalescent serum from Chinese patients hospitalized from January to April 2020 with no vaccination history (*n* = 24) or 1 dose of vaccine (*n* = 20) also exhibited reduced neutralization against BA.1 compared to wild type using a pseudovirus assay (43). A small number of specimens collected from Austrian patients with ancestral infection (March and April 2020 [*n* = 10]) had reduced neutralization of BA.1, using a focus-forming neutralization assay (44).

None of the specimens screened with the Abbott Quant assay had a value of ≥2 × 10^4^ BAU/mL, which has been previously associated with high-titer plasma against Omicron BA.1 (28). This is not unexpected, as convalescent plasma collected during earlier waves of the pandemic may have reduced efficacy against Omicron subvariants as they arise (45). However, this finding does not imply that the donors tested lacked protection against SARS-CoV-2 disease and death. Immunity to SARS-CoV-2 is complex and involves neutralizing antibodies, binding antibodies, antibody-dependent cellular cytotoxicity (46), complex mechanisms of cell-mediated immunity (47), and elements of innate immunity (48). Due to this complexity, there is still no wide consensus on correlations of protection to SARS-CoV-2 (16, 17). Apart from a potential role as a cutoff for high-titer convalescent plasma by convalescent plasma trials (28, 29), there is also no international consensus on the protective utility of the binding antibody value of ≥2 × 10^4^ BAU/mL (30, 49).

A full year of the pandemic would need to pass before the Canadian population developed high BAU per milliliter values. A larger Canadian seroprevalence study (10,000 to 40,000 specimens/month) first identified median BAU/mL levels of ≥2 × 10^4^ BAU/mL in February of 2022 after the emergence of Omicron. However, the low frequency of anti-N and high frequency of anti-S in the population suggests that high BAU per milliliter values were being driven by COVID-19 vaccination programs rather than natural infection (50). This study does not discriminate between the impacts of boosters or new bivalent vaccines. However, it is important to note the benefit of SARS-CoV-2 vaccines in reducing disease burden and death in the Canadian population, even in an environment dominated by Omicron (51–53). The rollout of SARS-CoV-2 vaccines in Canada can be seen as a success story, with 85% of Canadians receiving at least one dose and 82% receiving a primary series by 11 September 2022. However, some Canadians expressed antivaccine sentiments, lacking understanding of vaccines and herd immunity (54), and vaccine-hesitant individuals often expressed a preference for natural immunity (55).

This study has several additional caveats. Different cell culture conditions were used for wild type, Alpha, Beta, Gamma, and Delta than for Omicron. To account for this, the study focused on identifying the presence or absence of any neutralizing antibody capacity against SARS-CoV-2 VOCs. This study included a small number of specimens for the time from January 2021 to March 2021 used for PRNT_50_ (13). Due to the time taken to develop Omicron BA.1 PRNT_50_ assays, this study did not assess donor plasma for neutralization against later sublineages of BA.1, BA.2, BA.3, BA.4, BA.5, or recombinants that have circulated in Canada (56). It is also important to acknowledge that donor-declared histories of vaccination may be confounded by recall bias and may be incomplete (57). The collection of vaccination histories, as approved in the study ethics proposal, was also limited to the specimens used for PRNT_50_ and not linked to data broadly tested with the Abbott Quant assay.

Although this work relies on specimens collected early in the pandemic, it does have applicability to understanding humoral immunity in individuals who are partially vaccine hesitant (receiving less than a full series of wild-type SARS-CoV-2 vaccine) or completely vaccine hesitant (relying on immunity from an earlier infection with wild-type or Alpha SARS-CoV-2). Those individuals may have impaired humoral protection against Omicron BA.1 SARS-CoV-2 infection. Therefore, even in populations with high rates of SARS-CoV-2 infection, vaccination (including boosting with monovalent or bivalent vaccines) is an important strategy in reducing the burden of severe disease and death (58, 59). This protection is broad and ensures the safety of adults and children in the population from outcomes including intensive care admission and death, even when Omicron is dominant (51).

## MATERIALS AND METHODS

### Ethical considerations.

Institutional ethics board clearance for this project was received from the University of Alberta and the following institutions: Canadian Blood Services and Sinai Health, Toronto (Mount Sinai Hospital).

### CIHR Correlates of Immunity study participants and samples.

Canadian Blood Services collects retention EDTA plasma (Becton Dickson [BD], Mississauga, ON, Canada) specimens as previously described (12, 13, 32, 60). As previously described, this was a repeated cross-sectional design with random cross-sectional sampling of all available retention samples (*n* = 1,500/month) for a 12-month period from January, February, and March of 2021 (total *n* = 4,500) (20). Samples were then anonymized, aliquoted, transported to test sites, and then stored (−40 to −80°C) (12). A total of 4,453 retention specimens were available from January (*n* = 1,499), February (*n* = 1,465), and March (*n* = 1,489) for testing with the Abbott Quant assay.

### Donor SARS-CoV-2 vaccination history and linking to specific specimens.

During the donation screening process, all donors were asked if they received a SARS-CoV-2 vaccine in the past 3 months. This was standard practice at Canadian Blood Services, did not collect information on the vaccine producer, and was not linked to provincial vaccination records. Donor vaccine information focused on donors with specimens linked to PRNT_50_ neutralization assays (12).

### Specimens chosen for SARS-CoV-2 neutralization testing.

Specimens assessed for antibody neutralizing capacity of wild type and variant (Alpha, Beta, Gamma, Delta, and Omicron BA.1) were previously selected using a published tiered testing approach (12, 13).

### Definitions of evidence of anti-N positivity.

Serological evidence of anti-N positivity was defined as the presence of an anti-N signal by at least one of the Abbott Architect anti-N SARS-CoV-2 IgG assay or the Sinai Health anti-N assay (see previous publication [12]).

### PRNT_50_ assays: wild type and variants of concern.

Selected EDTA plasma specimens were used in PRNT_50_ experiments. Vero cell cultures were used for Wuhan wild type (hCoV-19/Canada/ON_ON-VIDO-01-2/2020, Global Initiative on Sharing All Influenza Data [GISAID, https://gisaid.org/] accession number EPI_ISL_425177) and variant of concern strains (Alpha [B.1.1.7], Beta [B.1.351], Gamma [P.1], and Delta [B.1.617.2]). Culture conditions for wild type, Alpha, Beta, Gamma, and Delta followed the experimental conditions previously described (12, 61). For Omicron PRNT_50_, experimental conditions varied only in that PRNT_50_ plates were incubated for 3 days prior to fixation with crystal violet-formaldehyde solution for at least 1 h. After rinsing with distilled water (dH_2_O), plates were air-dried, and plaques were counted on a lightbox (for the detailed PRNT_50_ procedure, please see Valcourt et al. [61] and Lin et al. [12]). The Omicron virus stock was a clinical isolate passaged in Vero E6 and TMPRSS2 cells, and next-generation sequencing (NGS) was used to confirm the Omicron BA.1 sequence.

### SARS-CoV-2 antibody testing using the Abbott Quant assay.

We tested 4,453 randomly selected retention specimens by using the Abbott Quant assay (Abbott Laboratories, Chicago, IL, USA) as per the manufacturer’s guidelines and as previously described (12). These specimens were not subjected to prior stratification based on anti-N, anti-RBD, or anti-S. Semiquantitative values (units per milliliter) generated by the Abbott Quant were converted to BAU per milliliter as described in a prior analysis (12, 20).

### Data storage and statistical analysis.

A study identification number was assigned by the information technology team at Canadian Blood Services. All samples were labeled with a study identification number, and all data were stored with this number. Researchers did not have access to the donor-identifying data. Data were stored using a password-protected Microsoft Excel (Redmond, WA, USA) spreadsheet. Descriptive data (median, 25th percentile, and 75th percentile), Fisher's exact test (two-sided), odds ratios, and 95% CIs were calculated with GraphPad Prism (version 9.2.0; GraphPad Software, Inc., San Diego, CA, USA) was used to analyze data. PRNT_50_ values were assessed for the presence (yes or no) of any measurable neutralizing response against wild type and variant (Alpha, Beta, Gamma, Delta, BA.1 Omicron) SARS-CoV-2.
